# Whole‐Body Metabolism and the Musculoskeletal Impacts of Targeting Activin A and Myostatin in Severe Osteogenesis Imperfecta

**DOI:** 10.1002/jbm4.10753

**Published:** 2023-05-07

**Authors:** Catherine L. Omosule, Dominique Joseph, Brooke Weiler, Victoria L. Gremminger, Spencer Silvey, Brittany N. Lafaver, Youngjae Jeong, Sandra Kleiner, Charlotte L. Phillips

**Affiliations:** ^1^ Department of Biochemistry University of Missouri Columbia Missouri USA; ^2^ Regeneron Pharmaceuticals New York USA; ^3^ Department of Child Health University of Missouri Columbia Missouri USA

## Abstract

Mutations in the COL1A1 and COL1A2 genes, which encode type I collagen, are present in around 85%–90% of osteogenesis imperfecta (OI) patients. Because type I collagen is the principal protein composition of bones, any changes in its gene sequences or synthesis can severely affect bone structure. As a result, skeletal deformity and bone frailty are defining characteristics of OI. Homozygous *oim/oim* mice are utilized as models of severe progressive type III OI. Bone adapts to external forces by altering its mass and architecture. Previous attempts to leverage the relationship between muscle and bone involved using a soluble activin receptor type IIB‐mFc (sActRIIB‐mFc) fusion protein to lower circulating concentrations of activin A and myostatin. These two proteins are part of the TGF‐β superfamily that regulate muscle and bone function. While this approach resulted in increased muscle masses and enhanced bone properties, adverse effects emerged due to ligand promiscuity, limiting clinical efficacy and obscuring the precise contributions of myostatin and activin A. In this study, we investigated the musculoskeletal and whole‐body metabolism effect of treating 5‐week‐old wildtype (Wt) and *oim*/*oim* mice for 11 weeks with either control antibody (Ctrl‐Ab) or monoclonal anti‐activin A antibody (ActA‐Ab), anti‐myostatin antibody (Mstn‐Ab), or a combination of ActA‐Ab and Mstn‐Ab (Combo). We demonstrated that ActA‐Ab treatment minimally impacts muscle mass in *oim*/*oim* mice, whereas Mstn‐Ab and Combo treatments substantially increased muscle mass and overall lean mass regardless of genotype and sex. Further, while no improvements in cortical bone microarchitecture were observed with all treatments, minimal improvements in trabecular bone microarchitecture were observed with the Combo treatment in *oim*/*oim* mice. Our findings suggest that individual or combinatorial inhibition of myostatin and activin A alone is insufficient to robustly improve femoral biomechanical and microarchitectural properties in severely affected OI mice. © 2023 The Authors. *JBMR Plus* published by Wiley Periodicals LLC on behalf of American Society for Bone and Mineral Research.

## Abstract 2

We investigated the impact of anti‐activin A and anti‐myostatin antibody treatment on bone and muscle properties in a severe mouse model of osteogenesis imperfecta. Treatment failed to substantially improve skeletal bone properties, although increases in muscle mass were observed when anti‐myostatin antibody was included in the treatment regimen.

## Introduction

Skeletal tissue provides balance, protects internal organs from traumatic injury, and is important for mobility. The primary protein constituent of bone tissue is type I collagen, a heterotrimeric molecule synthesized from the COL1A1 and COL1A2 genes. Defects in the structure and synthesis of type I collagen can result in a rare and heritable musculoskeletal disorder known as osteogenesis imperfecta (OI). OI is a connective tissue disorder associated with low bone mass, bone fragility, and skeletal deformity. Classical autosomal dominant type III OI is the most severe survivable form, with patients exhibiting significant progressive skeletal fragility and deformity.^(^ ^1^ ^)^ While there is currently no cure for OI, surgical rodding and bone antiresorptive medications are frequently used OI management therapies. These can improve mobility and increase bone mass but fail to be without adverse effects, necessitating the development of more effective OI treatment strategies.^(^ ^2^, ^3^, ^4^ ^)^ OI individuals also exhibit intrinsic muscle weakness, a phenomenon that likely exacerbates bone fragility given the intimate connection between bone and muscle strength.^(^ ^5^, ^6^, ^7^, ^8^ ^)^ Therefore, therapeutic options that address both bone and muscle weakness are highly sought after for treating OI.^(^ ^9^, ^10^ ^)^

Myostatin and activin A are members of the TGF‐β superfamily of proteins and are produced by muscle and bone cells, respectively. Both myostatin and activin A induce canonical signaling cascades through the activin type II and I receptor kinases (ActRIIB and Alk4/5/7), stimulating downstream intracellular Smad signaling.^(^ ^11^, ^12^, ^13^ ^)^ As a negative regulator of muscle mass, the absence of myostatin results in a double‐muscled phenotype characterized by substantial increases in muscle mass.^(^ ^14^, ^15^ ^)^ Pharmacologically induced myostatin deficiency also results in muscle hypertrophy in multiple species.^(^ ^16^, ^17^, ^18^, ^19^ ^)^

Activin A has metabolic roles in bone that are not yet well characterized.^(^ ^20^ ^)^ Previous studies suggested that activin A could spur osteoclast development in cultures and that the introduction of inhibin and the removal of activin A using soluble ActRIIA receptors restore bone formation and improve bone healing after fracture.^(^ ^21^, ^22^, ^23^ ^)^ Whereas there are mixed reports on the effects of activin A on osteoblasts, more recent studies suggest that activin A inhibits osteoblast‐controlled mineralization.^(^ ^20^, ^24^ ^)^

Deficiency in myostatin alone is sufficient to elicit muscle hypertrophy. However, in both rodents and primates, additive inhibition of activin A more potently induces muscle mass formation.^(^ ^18^ ^)^ In addition, ligand traps that target both myostatin and activin A are more effective at preventing bone loss and enhancing bone mass than inhibition of myostatin alone.^(^ ^25^, ^26^, ^27^ ^)^ This is because of the pleiotropic ability of these ligand traps to harness the power of the bidirectional and intimate biochemical and biomechanical relationship between muscle and bone.^(^ ^16^, ^19^, ^28^, ^29^ ^)^ Even so, ActRIIB ligand traps lower circulating serum levels of multiple ligands in addition to activin A and myostatin, obscuring the precise contributions of myostatin and activin A in postnatal muscle mass increase.^(^ ^30^ ^)^ Moreover, antagonistic effects like epistaxis, telangiectasia, and gingival bleeding, alongside the lack of consistent functional improvements with soluble ActRIIB molecule treatment in humans has generated concerns regarding the use of ActRIIB ligand traps.^(^ ^30^, ^31^, ^32^ ^)^

The *oim* mouse originally arose from a spontaneous nucleotide deletion in the C‐terminal end of the *Col1a2* gene, generating a mouse with severely compromised skeletal phenotype.^(^ ^33^ ^)^ Homozygote *oim/oim* mice have inherent muscle pathology, demonstrated by appreciably smaller hindlimb muscles and impaired muscle contractility force.^(^ ^7^ ^)^ *Oim*/*oim* mice also exhibit a progressively deforming skeletal phenotype, synonymous to type III human OI.^(^ ^34^ ^)^ Even so, ActRIIB ligand traps increase muscle mass and function, in addition to skeletal microarchitectural properties in male and female *oim/oim* mice.^(^ ^16^, ^19^, ^35^ ^)^

Currently, the impact of specific inhibition of activin A alone, myostatin alone, or both in the severe type III *oim*/*oim* mouse has not been explored. Thus, to begin to tease out the regulatory roles of activin A and myostatin in postnatal skeletal regulation in severe OI, we have examined the effect of anti‐activin A antibody alone, anti‐myostatin antibody alone, or combined anti‐activin A and anti‐myostatin antibody therapy on muscular and skeletal properties, as well as whole‐body metabolism in homozygote *oim*/*oim* mice.

## Materials and Methods

### Animals

All experiments were approved by the University of Missouri Animal Care and Use Committee (ACUC) and met the ARRIVE guidelines. All study mice had free access to food and water and were housed in an AAALAC facility with 12 hours alternating light and dark cycles. *Col1a2* ^*oim*/*oim*^ (*oim*/*oim*) mice and their Wt littermates were generated from heterozygous *oim* crosses (+/*oim* X + /*oim*) and maintained on the C57BL6 background.^(^ ^33^ ^)^

### Study design

Male and female Wt and *oim/oim* mice were randomly assigned to one of four treatment groups: control antibody (Ctrl‐Ab, Regn 1945), anti‐activin A antibody (ActA‐Ab, Regn2746), anti‐myostatin antibody (Mstn‐Ab, Regn647), or combination ActA‐Ab and Mstn‐Ab (Combo) (Regeneron Pharmaceuticals Inc., Tarrytown, New York).^(^ ^29^, ^36^ ^)^ Treatment of individual monoclonal antibodies (10 mg/kg of body weight) twice weekly was administered intraperitoneally from 5 to 16 weeks of age.^(^ ^36^ ^)^ Prior to each injection, body weights were recorded. Mice were humanely euthanized at 16 weeks of age, following 11 weeks of treatment.

### Quantitation of serum myostatin and activin a

To determine basal levels of circulating myostatin and activin A, blood was collected at the time of sacrifice from untreated 4‐month‐old male and female Wt and *oim*/*oim* mice by cardiac puncture and the serum isolated by centrifugation at 14,000 rpm for 15 minutes and stored at −80°C until assayed. Serum levels of myostatin and activin A were quantified using commercially available ELISA kits, the GDF‐8/Myostatin Quantikine ELISA Kit (DGDF80, R&D Systems, Minneapolis, Minnesota) and the Human/Mouse/Rat Activin A Quantikine ELISA Kit (DAC00B, R&D Systems), respectively. Samples and standards were performed in duplicate following the manufacturer's instructions. A standard curve was generated by plotting the absorbance and concentration values of the standards using a four‐parameter logistic curve fit (online data analysis tool, MyAssays Ltd.) according to the manufacturer's instructions.

### Body composition and whole‐body metabolic assessment

As previously reported for whole‐body metabolic assessments, mice were placed within PromethION metabolic cages to track their energy expenditure (EE), respiratory quotient, and activity levels between 14 and 16 weeks of age.^(^ ^36^ ^)^ Mice were individually housed for 3 days, with the first day considered an acclimatization period. Data were collected in diurnal (7 a.m. to 7 p.m.) and nocturnal (7 p.m. to 7 a.m.) cycles. EE, mean O_2_ consumption, and mean CO_2_ production were normalized to lean mass.

Following metabolic cage assessments, whole‐body composition in live mice was assessed using the Echo Magnetic Resonance Imaging (echoMRI) system. System tests and calibrations were performed prior to each daily set of analyses. For assessments, live mice were individually placed in the echoMRI body composition analyzer (E26‐242‐RMT, Echo Medical Systems, USA). Absolute and relative lean mass and fat mass were determined.

### Hindlimb tissue collection

Following sacrifice, left hindlimb gastrocnemius, quadriceps, tibialis anterior (TA), plantaris, soleus, and extensor digitorum longus (EDL) muscles were harvested and weighed. Skeletal calluses and fractures were recorded at the time of hindlimb muscle and long bone collection.

### Femoral microarchitecture

Excised right femora were cleaned of soft tissues, wrapped in gauze, and stored in 1× PBS at −20°C until micro–CT (μCT) analyses were conducted using the vivaCT 40 μCT scanner (SCANCO Medical AG, Bassersdorf, Switzerland) and the following parameters: 70kVp, 114 μA, 8 W X‐ray energy intensity, high‐resolution CT scan with 10‐μm isotropic voxel size (10 × 10 × 10 μm),^(^ ^37^ ^)^ and an integration time of 300 ms, as previously described.^(^ ^29^ ^)^

### Femoral biomechanical testing

After μCT, right femora were subjected to three‐point bend analyses using the Instron 5942 Universal testing System (Instron, Norwood, MA, USA) and BlueHill 3 Software version 3.53 (Illinois Tool Works Inc., Glenview, IL, USA). Bones were placed anteroposteriorly on support stands that were 9 mm apart. Testing was performed using a load cell with a maximum scale of 5 kg set at an automatic trigger force of 0.2 N and a constant speed of 5 mm/min until bone failure. Ultimate load, yield load, stiffness, postyield displacement, and work‐to‐fracture were determined from the load displacement curve using Microsoft Excel, as previously described.^(^ ^29^ ^)^

### Statistics

**Fig. 1:**
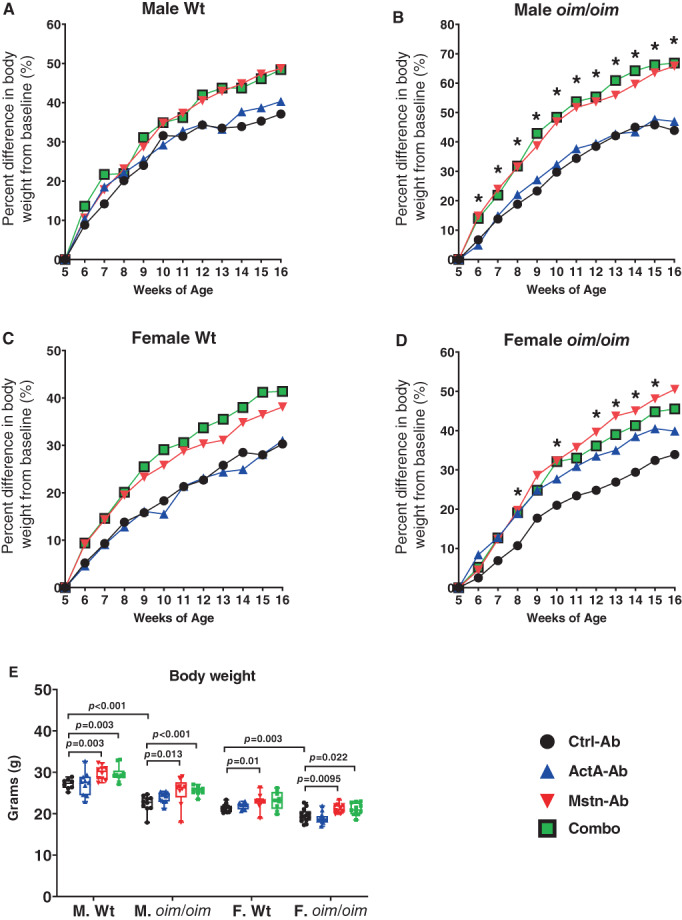
Percentage difference in body weight with treatment. Male Wt (*A*), male *oim/oim* (*B*), female Wt (*C*), and female *oim*/*oim* (*D*) mice were treated with control antibody (Ctrl‐Ab, black circle), anti‐activin A antibody (ActA‐Ab, blue triangle), anti‐myostatin antibody (Mstn‐Ab, red triangle), or combination anti‐activin A and anti‐myostatin antibodies (Combo, green square) from 5 to 16 weeks of age. Final body weights are presented in (*E*). Mean values are plotted (*A*–*D*); *n* = 7–12. *Significant at *p* ≤ 0.05 for Combo treatment (*A*–*D*) versus Ctrl‐Ab treatment. In €, *n* = 8–13; Data represent min and max box and whisker plot with all data points shown; *p* ≤ 0.1 are indicated, and *p* ≤ 0.05 is considered significant.

Statistical analysis and graphing were performed using SAS software (SAS Institute, Inc., Cary, NC, USA) and GraphPad Prism (GraphPad Software, San Diego, CA, USA). Weekly body weights (Fig. 1*A*–*D*) were analyzed as previously described, using the first‐order autoregressive AR (1) model.^(^ ^29^ ^)^ For all remaining analyses, nonparametric Mann–Whitney U tests were used to identify statistically significant differences at *p* ≤ 0.05. For all comparisons, *p* ≤ 0.1 is indicated in graphs.

## Results

### Basal concentrations of serum activin a and myostatin

Although previous studies demonstrated the efficacy of ActRIIB ligand traps in increasing musculoskeletal properties in *oim/oim* mice,^(^ ^16^, ^19^, ^35^ ^)^ it was crucial to establish the basal blood concentrations of activin A and myostatin in *oim/oim* mice in order to properly interpret the outcome measures of this study. We observed equivalent concentrations of serum myostatin and activin A in 4‐month‐old mice regardless of genotype or sex (Fig. S1), although female *oim*/*oim* mice appeared to have lower circulating serum myostatin concentrations, which did not reach statistical significance at *p* < 0.05.

### Casualties and calluses

During this study, two Wt mice (1 Mstn‐Ab; 1 ActA‐Ab) and 11 *oim*/*oim* mice (3 Mstn‐Ab; 5 ActA‐Ab, and 3 Ctrl‐Ab) died. One *oim/oim* mouse died of a compound fracture sustained 2 weeks into ActA‐Ab treatment, whereas another ActA‐Ab treated mouse was culled due to severe malocclusion. The remaining mice were found dead in their cages and were mostly 6 or 7 weeks of age at the time of their death. Cause of death was not investigated. Among the experimental mice that completed the study, 40 *oim/oim* male and female mice sustained at least one callused femur, tibia, or forelimb bone (Table 1). Most of the calluses observed were on the femurs (*n* = 34 mice). The distribution of limb calluses among the treatment groups appeared random, and we were unable to determine whether the calluses occurred prior to treatment initiation at 5 weeks of age or were acquired during treatment. Callused limb bones were excluded from all bone analyses.

**Table 1:** Number of *oim*/*oim* mice with at least one callused long bone (femur, tibia, and/or forelimb)

	Ctrl‐ab	ActA‐ab	Mstn‐ab	Combo
Male Wt	0 (8)	0 (10)	0 (10)	0 (10)
Female Wt	0 (11)	0 (9)	0 (10)	0 (9)
Male *oim/oim*	6 (10)	9 (11)	5 (10)	5 (9)
Female *oim/oim*	3 (13)	3 (12)	3 (10)	6 (11)

*Note*: Numbers in parentheses represent total number of mice per treatment group.

### Growth trend and body weights

Figure 1 illustrates the effect of treatment on body weight relative to starting body weight at 5 weeks of age. In male Wt mice, the Combo treatment failed to increase body weights to significant levels (Fig. 1*A*), whereas in male *oim/oim* mice, the Combo treatment resulted in considerable increases in body weight compared to Ctrl‐Ab‐treated littermates following 1 week of treatment (Fig. 1*B*). In Wt female mice, the Combo treatment failed to result in consequential changes in body weight relative to control antibody treatment, although in *oim/oim* female mice, substantial increases in body weight were first observed 3 weeks following treatment initiation (Fig. 1*C*,*D*).

At the end of the study period, at 16 weeks of age, both male and female control *oim*/*oim* mice had lower body weights relative to their Wt counterparts (−21.2% [*p* < 0.001] and − 9.4% [*p* = 0.003], respectively) (Fig. 1*E*). ActA‐Ab treatment failed to significantly increase body weights in study mice regardless of genotype or sex. Mstn‐Ab treatment increased overall body weights in male Wt mice (+10%, *p* = 0.003), male *oim/oim* mice (+13.4%, *p* = 0.013), female Wt mice (+7.5%, *p* = 0.01), and female *oim*/*oim* mice (+8.5%, *p* = 0.01). The Combo treatment also increased body weights in male Wt mice (+8.9%, *p* = 0.003), male *oim/oim* mice (+14.1%, *p* < 0.001), and female *oim/oim* mice (+7.8%, *p* = 0.022) but failed to reach statistical significance in female Wt mice.

### Assessment of body composition

**Fig. 2:**
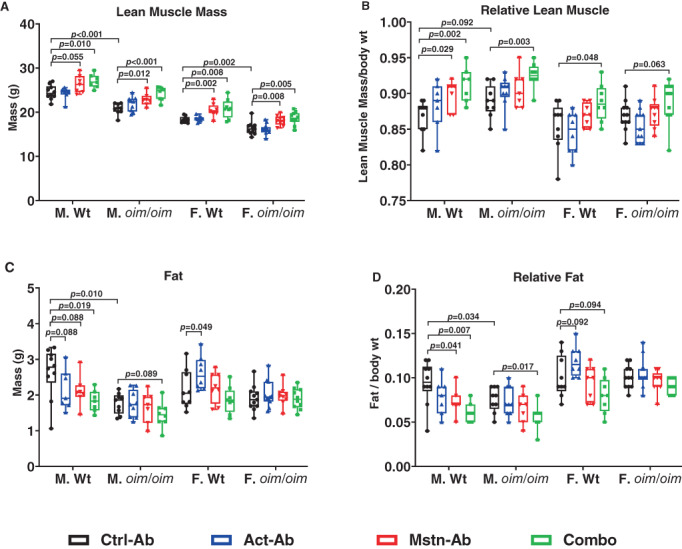
Body composition analyses of Wt and *oim*/*oim* mice treated twice weekly with 10 mg/kg of body weight of control antibody (Ctrl‐Ab, black circle), anti‐activin A antibody (ActA‐Ab, blue triangle), anti‐myostatin antibody (Mstn‐Ab, red triangle), or combination anti‐activin A and anti‐myostatin antibodies (Combo, green square) at 5–16 weeks of age. (*A*) Absolute lean mass (g), (*B*) relative lean mass (mg/g), (*C*) absolute fat mass (g), and (*D*) relative fat mass (mg/g). Data represent min and max box and whisker plot with all data points shown; *n* = 9–15 mice per group; *p*‐values < 0.1 are indicated, and *p* ≤ 0.05 is considered significant.

EchoMRI body composition analyses revealed lower absolute lean mass in Ctrl‐Ab‐treated male and female *oim/oim* mice (Fig. 2*A*) compared to Ctrl‐Ab treated Wt mice. Ctrl‐Ab‐treated male *oim/oim* mice also had lower absolute and relative fat mass as compared to Ctrl‐Ab‐treated male Wt mice (Fig. 2*C*,*D*).

ActA‐Ab treatment only increased absolute fat mass in female Wt mice. Mstn‐Ab treatment increased absolute lean mass in mice regardless of sex or genotype, as well as relative lean mass in male Wt mice only, while decreasing relative fat mass in male Wt mice only. The Combo treatment consistently increased absolute lean mass in mice regardless of sex or genotype, increased relative lean mass in male Wt and *oim*/*oim* mice as well as female Wt, and decreased absolute fat mass in male Wt mice, as well as relative fat mass in male Wt and *oim/oim* mice. The observation that the Combo treatment more robustly impacted muscle and fat masses in mice emphasizes the combined roles of myostatin and activin A in postnatal muscular and adipose tissue regulation in mice.^(^ ^18^ ^)^

Neither Mstn‐Ab nor ActA‐Ab effected significant changes in absolute or relative inguinal tissue, gonadal fat, or brown adipose tissue (BAT) mass in study mice (Fig. S2). However, the Combo treatment resulted in decreases in individual fat masses, some of which were significant, as observed in the gonadal fat mass of female *oim*/*oim* mice as well as the relative BAT of male *oim*/*oim* mice and female Wt mice.

### Hindlimb muscle weights at 16 weeks of age

**Fig. 3:**
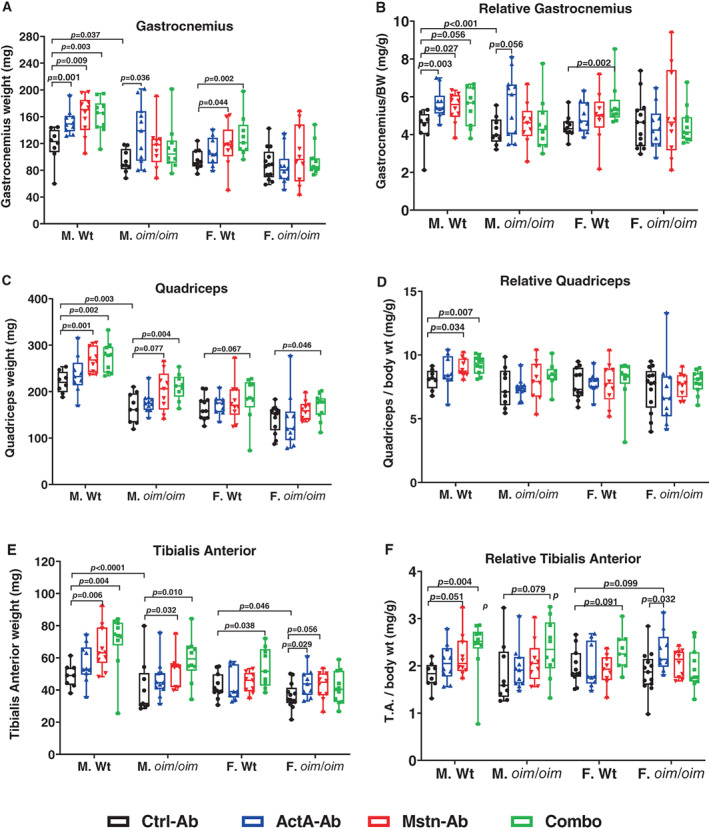
Hindlimb muscle weights of Wt and *oim*/*oim* mice treated twice weekly with 10 mg/kg of control antibody (Ctrl‐Ab, black circle), anti‐activin A antibody (ActA‐Ab, blue triangle), anti‐myostatin antibody (Mstn‐Ab, red triangle), or combination anti‐activin A and anti‐myostatin antibodies (Combo, green square) from 5 to 16 weeks of age. (*A*) Absolute gastrocnemius, (*B*) relative gastrocnemius, (*C*) absolute quadriceps, (*D*) relative quadriceps, (*E*) tibialis anterior (TA), and (*F*) TA weights. Data represent min and max box and whisker plot with all data points shown; *n* = 8–13 mice per group; *p*‐values ≤ 0.1 are indicated, and *p* ≤ 0.05 is considered significant.

At 16 weeks of age, the hindlimb muscle weights of control *oim*/*oim* male mice were considerably lower for the absolute and relative gastrocnemius (Fig. 3*A*,*B*), absolute quadriceps (Fig. 3*C*), and TA (Fig. 3*E*) muscles relative to equivalent muscle groups in control Wt male mice. Female control *oim*/*oim* mice also had lower absolute TA muscle masses relative to their Wt counterparts (*p* = 0.066).

ActA‐Ab treatment increased gastrocnemius muscle mass in male Wt mice (absolute and relative) and male *oim/oim* mice (absolute), as well as absolute and relative TA muscle masses in female *oim/oim* mice.

In male Wt mice, Mstn‐Ab treatment increased gastrocnemius, quadriceps, and TA muscle weights, whereas in female Wt mice, Mstn‐Ab treatment only increased absolute gastrocnemius muscle weights. Among *oim*/*oim* mice, Mstn‐Ab treatment increased absolute gastrocnemius and TA muscle mass in male and resulted in a trend toward increasing absolute TA muscle in female mice.

The Combo treatment increased absolute weights for all evaluated hindlimb muscles in Wt mice, although not reaching significance in Wt female quadriceps muscle. Relative gastrocnemius and relative TA muscle masses were also increased in female and male Wt mice, respectively. Among *oim*/*oim* mice, the Combo treatment increased absolute quadriceps muscle weights regardless of sex, but only increased absolute TA muscle weights in male *oim/oim* mice.

### Heart and spleen weights

Control male Wt mice had absolute heart and spleen weights similar to those of control male *oim/oim* mice. However, their relative heart weights were significantly lower than those of male *oim/oim* relative heart weights (Fig. S3). Control female Wt mice also had absolute heart weights similar to those of control female *oim*/*oim* mice. Yet, control female *oim*/*oim* relative heart, absolute spleen, and relative spleen weights were elevated compared to their control female Wt tissue counterparts.

Neither ActA‐Ab nor Mstn‐Ab treatment impacted heart or spleen weights in all study mice. However, the Combo treatment resulted in decreased absolute and relative spleen weights in male *oim/oim* mice and only decreased relative spleen weights in female *oim/oim* mice.

### Assessment of whole‐body metabolism and energy expenditure

Metabolic chamber analyses revealed that control female *oim*/*oim* mice exhibited a different energy/metabolic profile relative to control female Wt mice (Fig. S4). EE and mean oxygen consumption (VO_2_) were lower among female *oim*/*oim* mice diurnally relative to Wt female mice. Male *oim*/*oim* mice displayed a trend toward lower VO_2_, although the difference was not significant at *p* < 0.05. Mean CO_2_ production and mean respiratory quotient (RQ) were equivalent between sex‐matched Wt and *oim*/*oim* mice.

ActA‐ab treatment alone resulted in the most significant changes in energy and metabolic profiles among study mice. While WT mice were unaffected by ActA‐Ab treatment, among *oim*/*oim* mice, female mice displayed higher EE and higher average VO_2_ consumption and VCO_2_ release during both the day and night cycles, as well as increased VO_2_ among male *oim*/*oim* mice during the night. Mstn‐Ab did not impact metabolic and energy levels, while the Combo treatment only increased EE and VO_2_ consumption in female *oim*/*oim* mice during the diurnal cycles.

Multidimensional beam break assessments revealed that control Wt mice had activity levels equivalent to those of control *oim*/*oim* mice (Fig. S5). Minimal treatment effects were observed. ActA‐Ab treatment increased the daytime activity level of male Wt mice relative to their control littermates. However, neither Mstn‐Ab nor Combo treatment altered activity levels.

### Trabecular bone microarchitecture

**Fig. 4:**
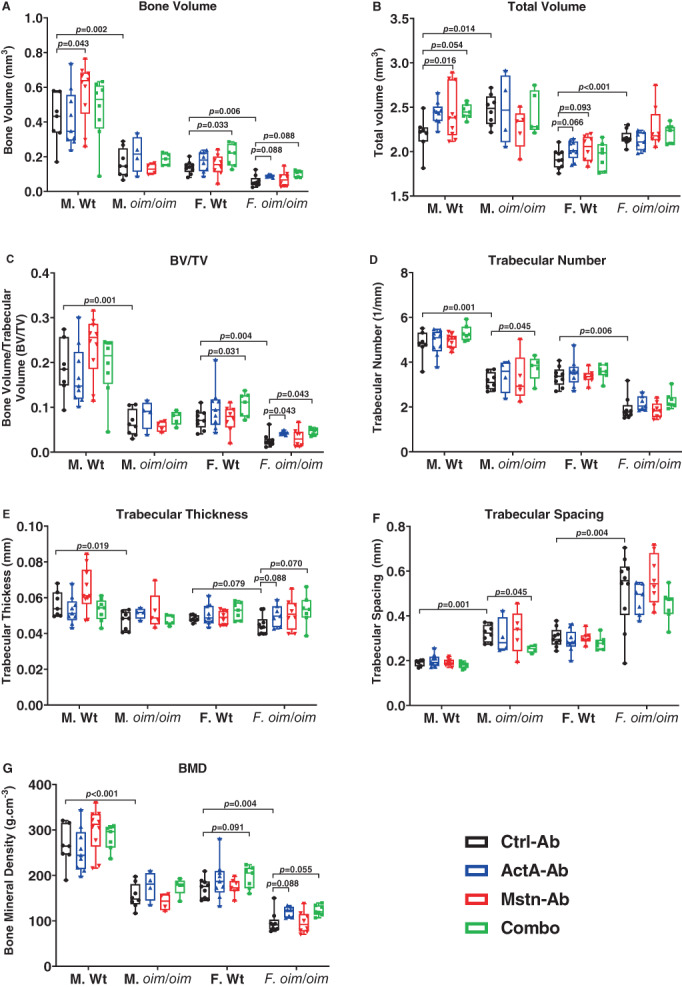
Femoral trabecular bone properties of Ctrl‐Ab‐, ActA‐Ab‐, Mstn‐Ab‐, and Combo‐treated Wt and *oim*/*oim* mice. (*A*) Bone volume (BV), (*B*) total volume (TV), (*C*) bone volume fraction (BV/TV), (*D*) trabecular number (Tb.N), (*E*) trabecular thickness (Tb.th), (*F*) trabecular spacing (Tb.Sp), and (*G*) bone mineral density (BMD). Mice were treated twice weekly with 10 mg/kg of control antibody (Ctrl‐Ab, black circle), anti‐activin A antibody (ActA‐Ab, blue triangle), anti‐myostatin antibody (Mstn‐Ab, red triangle), or combination anti‐activin A and anti‐myostatin antibodies (Combo, green square) from 5 to 16 weeks of age. Data represent min and max box and whisker plot with all data points shown; *n* = 4–9 mice per group; *p*‐values ≤ 0.1 are indicated, and *p* ≤ 0.05 is considered significant.

The outcomes of this study corroborated previous reports of inferior skeletal bone quality in homozygous *oim*/*oim* mice relative to Wt mice.^(^ ^34^, ^35^, ^38^ ^)^ Femoral trabecular bone volume (BV, Fig. 4*A*), BV fraction (BV/TV, Fig. 4*C*), trabecular number (Tb.N, Fig. 4*D*), and bone mineral density (BMD, Fig. 4*G*) were decreased in control *oim*/*oim* mice relative to sex‐matched control Wt littermates, whereas total volume (TV, Fig. 4*B*) and trabecular spacing (Tb.Sp, Fig. 4*F*) were increased. Trabecular thickness (Tb.th, Fig. 4*E*) was decreased in control *oim/oim* mice relative to control Wt mice, but only reached significance in males.

ActA‐Ab treatment increased BV/TV in female *oim*/*oim* mice and demonstrated trends toward increasing BV in female *oim*/*oim* mice, increasing TV in female Wt mice as well as increasing Tb.th and BMD in female *oim*/*oim*, although the differences did not reach significance. Mstn‐Ab treatment only increased TV and BV in male Wt mice. The Combo treatment did not significantly impact the femoral bone properties of male Wt mice, although it increased Tb.N and decreased Tb.Sp in male *oim*/*oim* mice. However, the Combo treatment increased BV and BV/TV in female WT mice and increased BV/TV in female *oim*/*oim* mice.

### Cortical bone microarchitecture

**Fig. 5:**
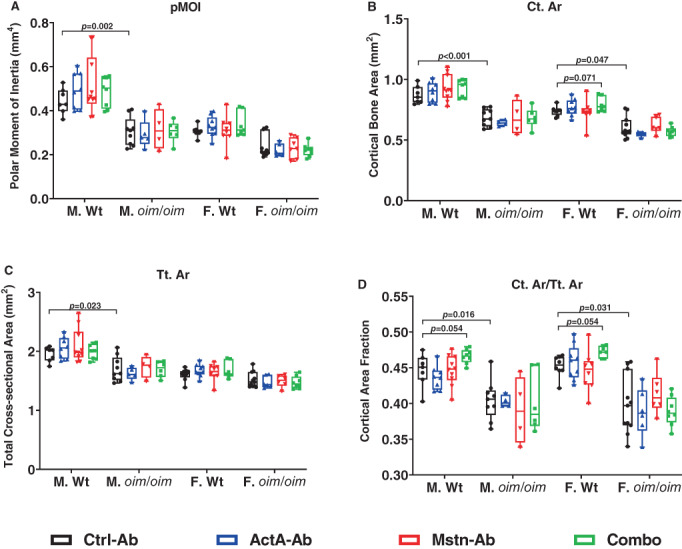
Cortical bone microarchitecture of Ctrl‐Ab, ActA‐Ab, Mstn‐Ab and Combo‐treated Wt and *oim*/*oim* mice. (*A*) Polar moment of inertia (pMOI), (*B*) cortical bone area (Ct.Ar), (*C*) total cross‐sectional area (Tt.Ar), (*D*) cortical bone fraction (Ct.Ar/Tt.Ar), and (*E*) length of right femur. Mice were treated twice weekly with 10 mg/kg of control antibody (Ctrl‐Ab, black circle), anti‐activin A antibody (ActA‐Ab, blue triangle), anti‐myostatin antibody (Mstn‐Ab, red triangle), or combination anti‐activin A and anti‐myostatin antibodies (Combo, green square) from 5 to 16 weeks of age. Data represent min and max box and whisker plot with all data points shown; *n* = 4–11 mice per group; *p*‐values < 0.1 are indicated, and *p* ≤ 0.05 is considered significant.

MicroCT analyses corroborated the substantial genotype‐based differences in femoral cortical bone properties. Control *oim*/*oim* mice had lower polar moment of inertia (pMOI, Fig. 5*A*), cortical bone area (Ct. Ar, Fig. 5*B*), total cross‐sectional area (Tt.Ar, Fig. 5*C*, in males only) and cortical area fraction (Ct.Ar/Tt.Ar, Fig. 5*D*) relative to age‐ and sex‐matched control Wt mice.

Cortical bone parameters remained unaltered with all treatment modalities in study mice regardless of sex or genotype, although the Combo treatment exhibited a trend toward increasing Ct.Ar in Wt female mice and Ct.Ar/Tt.Ar in Wt male and female mice.

### Bone biomechanical properties

**Fig. 6:**
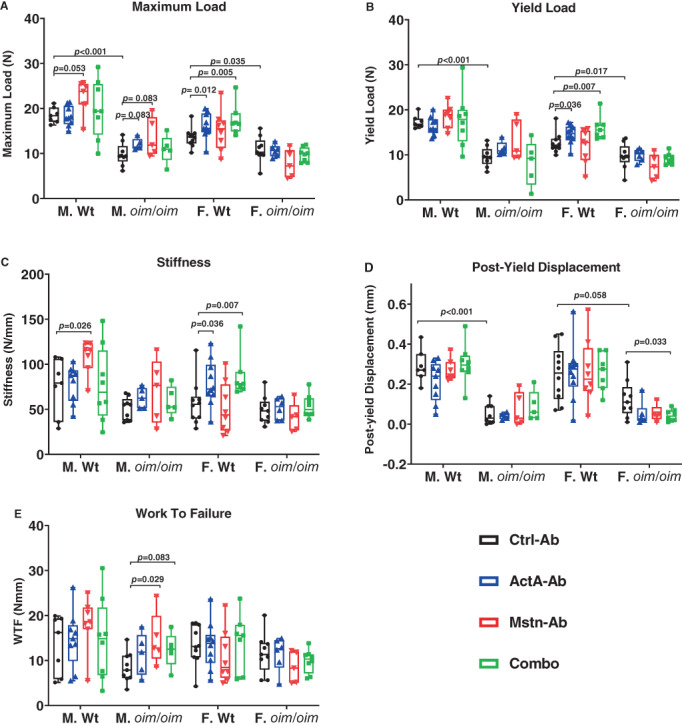
Bone biomechanical properties of Ctrl‐Ab‐, ActA‐Ab‐, Mstn‐Ab‐, and Combo‐treated Wt and *oim*/*oim* mice. (*A*) Maximum load, (*B*) yield load, (*C*) stiffness, (*D*) postyield displacement, and (*E*) work‐to‐fracture (WTF). Mice were treated twice weekly with 10 mg/kg of control antibody (Ctrl‐Ab, black circle), anti‐activin A antibody (ActA‐Ab, blue triangle), anti‐myostatin antibody (Mstn‐Ab, red triangle), or combination anti‐activin A and anti‐myostatin antibodies (Combo, green square) from 5 to 16 weeks of age. Data represent min and max box and whisker plot with all data points shown; *n* = 5–10 mice per group; *p*‐values < 0.1 are indicated, and *p* ≤ 0.05 is considered significant.

Femurs from control *oim*/*oim* mice had lower maximum load (males and females, Fig. 6*A*), yield load (males and females, Fig. 6*B*), and postyield displacement (males and females [*p* = 0.058], Fig. 6*D*). Stiffness (Fig. 6*C*) and work‐to‐failure (Fig. 6*E*) were equivalent among sex‐matched littermates.

ActA‐Ab treatment increased maximum load, yield load, and stiffness in female Wt mice. Mstn‐Ab only increased work‐to‐failure in male *oim*/*oim* mice. Femoral biomechanical changes with the Combo treatment were only observed in female Wt mice, which exhibited increases in maximum load, yield load, and stiffness, as well as in female *oim*/*oim* mice, which displayed lower postyield displacement.

## Discussion

In this study, we treated severe type III homozygous *oim*/*oim* with anti‐activin A antibody (ActA‐Ab) alone, anti‐myostatin antibody (Mstn‐Ab) alone, or in concert (Combo) and in this article reported their impact on muscle, bone, and whole‐body metabolic parameters. The Combo treatment and the Mstn‐Ab treatment alone were effective at increasing lean mass in mice compared to control antibody or ActA‐Ab treatments alone. The Combo treatment was effective at decreasing relative fat mass compared to treatment with either Mstn‐Ab or ActA‐Ab alone. The effects of Combo or Mstn‐Ab treatment were further reflected by the substantial increases in the gastrocnemius, quadriceps, and TA muscle weights, whereas ActA‐Ab treatment resulted in increased weights of only a select group of muscles based on sex and genotype. We also observed minimal treatment effects of ActA‐Ab, Mstn‐Ab, and Combo on femoral cortical bone microarchitecture, with inconsistent increases in femoral trabecular bone microarchitecture and biomechanics among the different genotypes and sex of mice. Lastly, we observed that young adult female *oim/oim* mice exhibited features suggestive of altered EE and metabolism including decreased energy mean EE and mean oxygen (VO_2_) consumption compared to sex‐matched Wt littermates.

In a previous study, we demonstrated that male and female mice, independently of genotype (i.e. Wt, +/*G610C*, or *oim*/*oim*), exhibited +20% and + 24% increases in body weight, respectively, when treated with the soluble activin receptor IIB (sActRIIB) decoy molecule.^(^ ^16^ ^)^ The sActRIIB decoy molecule binds multiple ActRIIB ligands, including activin A and myostatin. When monoclonal antibody therapy to two specific ActRIIB ligands are utilized, as was done in this study, we observed striking similarities and dissimilarities to previously reported sActRIIB‐mFc treatments. The combined inhibition of activin A and myostatin (Combo) yielded + 7.8% to +14.1% increases in body weight, which were equivalent to changes induced by Mstn‐Ab treatment alone (i.e., 7.5% to 13.4%). ActA‐Ab treatment did not result in statistically significant increases in body weight among study mice.

Latres and colleagues previously showed that treatment with combined anti‐activin A and anti‐myostatin antibodies increased TA muscle mass in SCID mice by +43.9% relative to anti‐myostatin antibody, akin to the increases engineered by ActRIIB.hFc treatment (+48.8%).^(^ ^18^ ^)^ In the present study, Mstn‐Ab significantly increased TA muscle weights in male Wt and *oim*/*oim* mice by +34.6% and + 27.5%, respectively, while the Combo treatment yield increased of +43% and + 42.8% in male Wt and male *oim*/*oim* mice, respectively. We also observed consistent increases in the weights of multiple muscle groups with the Combo treatment, an observation likely attributable to muscle fiber hypertrophy.^(^ ^16^ ^)^ Thus, inhibiting both activin A and myostatin (i.e., Combo treatment) had an additive impact on increasing muscle weights, giving support to the contributory role of activin A in murine postnatal muscle development.^(^ ^16^, ^28^, ^36^ ^)^

OI patients are more likely to have heart disease and cardiac complications due to abnormal or reduced type I collagen in heart tissue and valves.^(^ ^39^ ^)^ In support of this, *oim*/*oim* mouse hearts exhibited morphological alterations like lower collagen content and higher passive inflation rates.^(^ ^40^ ^)^ The *Aga2* mouse model of OI, which is caused by a dominant frameshift mutation in the C‐terminal region of the *Col1a1* gene, exhibits cardiac dysfunction, which is characterized by ventricular hypertrophy and defective myocardial matrix.^(^ ^41^ ^)^ While our study did not investigate heart morphology and function, we observed larger relative heart weights in male *oim*/*oim* mice and a trend toward increased relative heart weights in female mice compared to their sex‐matched Wt littermates. Importantly, our antibody treatment strategies (i.e., Mstn‐Ab or Combo) did not alter heart masses, in line with the observation that myostatin‐null mice do not exhibit a cardiac phenotype.^(^ ^42^ ^)^

We also observed a higher ratio of spleen to body weight, as well as a trend toward increasing absolute spleen weights in female *oim*/*oim* mice relative to female Wt mice. This is consistent with the observation that both male and female o*im*/*oim* mice exhibit splenomegaly between 5 and 20 weeks of age, a phenotype associated with chronic inflammation.^(^ ^43^ ^)^ Latres and colleagues revealed that combined monoclonal anti‐myostatin and anti‐activin A antibody therapy did not alter spleen weights in Wt mice,^(^ ^18^ ^)^ which was not observed in the *G610C* OI mouse model, where the Combo treatment decreased spleen weights in all +/*G610C* mice.^(^ ^36^ ^)^

In this study, we observed main treatment effects with the Combo treatment, which decreased both the absolute and relative spleen weights in male *oim*/*oim* mice, as well as the relative spleen weight in female *oim*/*oim* mice. The extent to which decreasing spleen weights and, by extension, decreasing inflammation impact skeletal health was not directly assessed in this study. Nevertheless, in a previous study, decreasing the concentration of circulating tumor necrosis factor α (TNFα) with concomitant decreases in spleen weights in female *oim*/*oim* mice failed to improve the OI skeletal phenotype,^(^ ^43^ ^)^ suggesting that the observed reduction in spleen weights with Combo treatment in this study likely had minimal impacts on skeletal features.^(^ ^43^ ^)^

The finding that female *oim*/*oim* mice exhibit a phenotype that is suggestive of dysregulation in whole‐body metabolism is particularly interesting. Although its clinical relevance is not completely understood, these findings are a call to action for more research into the relationship between whole‐body energy metabolism and OI pathogenesis. Young OI patients exhibit features reminiscent of a metabolic phenotype.^(^ ^44^ ^)^ Further, *Col1a1* ^*Jrt/+*^ mice also display prepubertal metabolic phenotypes.^(^ ^45^ ^)^ In this study, *oim*/*oim* mice were assessed between 14 and 16 weeks of age, when they are considered adult mice. We did not assess the age of onset of this metabolic phenotype, and the significance of its presence in female mice begs further investigation. As this phenomenon appears independent of physical activity levels, which were equivalent across sex and genotype in our study cohort.

In previous studies, male and female +/*G610C* mice were shown to have energy, metabolic, and activity levels similar to those of their Wt counterparts.^(^ ^36^ ^)^ Both *Col1a1* ^*Jrt*/+^ and *oim/oim* mice are severe models of human OI, while the *G610C* model reflects mild to moderately severe human OI. Therefore, we postulate that the stresses of repeated fractures, inflammation, skeletal deformity, functional impairment, and, perhaps, cellular stress^(^ ^34^, ^45^, ^46^ ^)^ likely impact energy and substrate utilization in these severe (*oim*/*oim* and *Col1a1* ^*Jrt*/+^) murine OI models relative to the milder OI models.

Additionally, we assessed femoral bone microarchitecture after euthanasia. Forty of 110 *oim/oim* mice had at least one healed callus in their femur, tibia, or forelimb bones, whereas Wt mice possessed no calluses. Unfortunately, we were unable to determine whether these calluses occurred prior to treatment initiation at 5 weeks of age or if they were acquired during treatment.

Jeong and colleagues previously reported three‐ to fourfold increases in femoral trabecular cortical bone microarchitecture in *oim*/*oim* mice treated with sActRIIB‐mFc for 8 weeks, although no changes in cortical bone parameters or biomechanical properties were observed.^(^ ^35^ ^)^ In the present study, femoral cortical bone properties remained unchanged regardless of sex, genotype, or treatment. Femoral trabecular bone properties were also minimally enhanced in *oim*/*oim* mice and female Wt mice with Combo treatment. Furthermore, femoral bone biomechanical properties of *oim*/*oim* mice remained largely unchanged with treatment. The absence of significant cortical and biomechanical skeletal improvements with ActA‐Ab, Mstn‐Ab, and Combo treatments mirrors the outcomes of previous investigations by Tauer and colleagues, in which Ace‐2494, an activin A‐/myostatin‐neutralizing antibody, stimulated increases in muscle mass and bone length but failed to enhance bone properties in the severe *Col1a1* ^*Jrt/+*^ mouse model, which, like the *oim/oim* mouse, spontaneously fractures and exhibits moderate to severe OI phenotypes.^(^ ^28^, ^47^ ^)^

Strontium ranelate therapy, anti‐sclerostin antibody therapy, TGF‐β antibody therapy, anti‐receptor activator of NF‐κB ligand antibody therapy, and activin A and myostatin inhibition therapies have all emerged out of a need to find more suitable OI management therapies.^(^ ^28^, ^29^, ^35^, ^38^, ^48^, ^49^, ^50^, ^51^, ^52^, ^53^ ^)^ It is evident from these and other studies that the key dependent variables for response to therapy are OI disease severity and disease gene variant. Murine models of severe OI (*Col1a1* ^*Jrt*/+^ and *oim*/*oim*) appear less amenable to therapy, whereas the models of mild to moderate OI (+/*oim*, +/*G610C*) tend to be more responsive,^(^ ^35^, ^50^, ^53^ ^)^ with a caveat that there are limited studies that compare treatment strategies across multiple OI variants.

The *oim*/*oim* bone exhibits a random distribution and organization of mineral crystals and has lower inelastic deformation.^(^ ^54^ ^)^ These features likely restrict the ability of antibody treatments to restore femoral and microarchitectural integrity. Nevertheless, the *oim*/*oim* bone retains the capacity to respond to muscle force when challenged,^(^ ^55^ ^)^ which, contrary to the observations of this study, were hypothesized to improve skeletal properties. Thus, it is likely that more aggressive treatment approaches that synergize the Combo treatment with therapies that are also aimed at restoring bone microarchitecture and cellular homeostasis may be successful.

The absence of serum myostatin and activin A concentrations at the start of therapy at 5 weeks old, which may have been different from concentrations at 4 months old and are shown in Fig. S1, is one of this study's limitations. Also, due to the minimal impact of ActA‐Ab, Mstn‐Ab, and Combo treatments on the bone microarchitecture and strength, further histological analyses of skeletal remodeling were not justifiable.

In conclusion, in this study, monoclonal anti‐activin A antibody alone, anti‐myostatin antibody alone, or combined monoclonal anti‐myostatin and anti‐activin A antibody treatments failed to generate significant phenotypic improvements in the *oim*/*oim* skeleton. This suggests that postnatal activin A and myostatin inhibition therapies alone, like sActRIIB‐mFc therapy for severe OI, are ineffective for treating severe OI. Nonetheless, there is optimism that new therapeutics that address both mechanistic musculoskeletal elements and cell stress reduction^(^ ^56^ ^)^ will have more significant therapeutic effects in severe OI.

## Author Contributions

**Catherine L Omosule:** Conceptualization; data curation; formal analysis; investigation; methodology; project administration; supervision; visualization; writing – original draft; writing – review and editing. **Dominique Joseph:** Data curation; formal analysis; investigation; project administration; supervision; visualization; writing – original draft; writing – review and editing. **Brooke Weiler:** Formal analysis; investigation; project administration; supervision; visualization; writing – review and editing. **Victoria L Gremminger:** Conceptualization; data curation; investigation; methodology; project administration; supervision; visualization; writing – review and editing. **Spencer Silvey:** Formal analysis; investigation; project administration; supervision; visualization; writing – review and editing. **Brittany N Lafaver:** Formal analysis; investigation; project administration; supervision; visualization; writing – review and editing. **Youngjae Jeong:** Conceptualization; methodology; writing – review and editing. **Sandra Kleiner:** Conceptualization; resources; writing – review and editing. **Charlotte Longacre Phillips:** Conceptualization; data curation; funding acquisition; methodology; project administration; resources; software; supervision; writing – original draft; writing – review and editing.

## Disclosures

Regn647, Regn2476, and Regn1945 were provided by Dr. Sandra Kleiner, an employee and shareholder of Regeneron Pharmaceuticals Inc. All other authors have stated they have nothing to disclose.

### Peer Review

The peer review history for this article is available at https://www.webofscience.com/api/gateway/wos/peer-review/10.1002/jbm4.10753.

## Supporting information


**Fig. S1.** Serum ELISA results comparing the expression of (*A*) myostatin (Mstn) and (*B*) activin A (ActA) in 4‐month‐old male and female Wt and *oim/oim* mice. No statistical difference was observed. *n* = 7–13.

**Fig. S2.** Heart and spleen weights of Wt and *oim*/*oim* mice treated twice weekly with 10 mg/kg of control antibody (Ctrl‐Ab, black circle), anti‐activin A antibody (ActA‐Ab, blue triangle), anti‐myostatin antibody (Mstn‐Ab, red triangle), or combination anti‐activin A and anti‐myostatin antibodies (Combo, green square) from 5 to 16 weeks of age. (*A*) Absolute heart weight (mg), (*B*) relative heart weight (mg/g), (*C*) absolute spleen weight (mg), and (*D*) relative spleen weight (mg/g). Data represent min and max box and whisker plot with all data points shown; *n* = 8–13 mice per group; *p*‐values ≤ 0.1 are indicated and *p* ≤ 0.05 is considered significant.

**Fig. S3.** Fat masses of Wt and *oim*/*oim* mice treated twice weekly with 10 mg/kg of control antibody (Ctrl‐Ab, black circle), anti‐activin A antibody (ActA‐Ab, blue triangle), anti‐myostatin antibody (Mstn‐Ab, red triangle), or combination anti‐activin A and anti‐myostatin antibodies (Combo, green square) from 5 to 16 weeks of age. (*A*) Absolute inguinal fat (inguinal tissue, mg), (*B*) relative inguinal fat (relative inguinal tissue, mg/g), (*C*) absolute gonadal fat (mg), (*D*) relative gonadal fat (mg/g), (*E*) absolute brown adipose tissue (BAT, mg), and (*F*) relative BAT (mg/g). Data represent min and max box and whisker plot with all data points shown; *n* = 4–10 mice per group; *p*‐values ≤ 0.1 are indicated, and *p* ≤ 0.05 is considered significant.

**Fig. S4.** Indirect calorimetry to assess energy status of Ctrl‐Ab‐, ActA‐Ab‐, Mstn‐Ab‐, and Combo‐treated Wt and *oim*/*oim* mice. (*A*) Mean energy expenditure (day cycle, kcal/h), (*B*) mean energy expenditure (night cycle, kcal/h), (*C*) mean O_2_ consumption (day cycle, mL/min), (*D*) mean O_2_ consumption (night cycle, mL/min), (*E*) mean CO_2_ production (day cycle, mL/min), (*F*) mean CO_2_ production (night cycle, mL/min), (*G*) mean respiratory quotient (day cycle), and (*H*) mean respiratory quotient (night cycle). Mice were treated twice weekly with 10 mg/kg of control antibody (Ctrl‐Ab, black circle), anti‐activin A antibody (ActA‐Ab, blue triangle), anti‐myostatin antibody (Mstn‐Ab, red triangle), or combination anti‐activin A and anti‐myostatin antibodies (Combo, green square) from 5 to 16 weeks of age. Data represent min and max box and whisker plot with all data points shown; *n* = 2–6. *p*‐values ≤ 0.1 are indicated, and *p* ≤ 0.05 is considered significant.

**Fig. S5.** Activity levels of Ctrl‐Ab‐, ActA‐Ab‐, Mstn‐Ab‐, and Combo‐treated Wt and *oim*/*oim* mice. Multidimensional beam breaks during (*A*) day and (*B*) night cycles are calculated from the averages of movement along the *x*‐, *y*‐, and *z*‐axes. Mice were treated twice weekly with 10 mg/kg of control antibody (Ctrl‐Ab, black circle) or a combination of activin A and myostatin antibodies (Combo, green square) from 5 to 16 weeks of age. Data represent min and max box and whisker plot with all data points shown; *n* = 5–10; *p*‐values ≤ 0.1 are indicated, and *p* ≤ 0.05 is considered significant.
