# Supplementary material for: Whole‐Body Metabolism and the Musculoskeletal Impacts of Targeting Activin A and Myostatin in Severe Osteogenesis Imperfecta
Source: JBMR Plus. 2023 May 7;7(7):e10753. doi: 10.1002/jbm4.10753 (PMC10339096; doi:10.1002/jbm4.10753)
Supplement: Supplementary file 1 — Fig. S1. Serum ELISA results comparing the expression of (A) myostatin (Mstn) and (B) activin A (ActA) in 4‐month‐old male and female Wt and oim/oim mice. No statistical difference was observed. n = 7–13. [file JBM4-7-e10753-s003.docx]

**Supplemental Figure 1**

Serum ELISA results comparing the expression of A) myostatin (Mstn) and B) Activin A (ActA) in 4-month-old male and female Wt and oim/oim mice. No statistical difference was observed. n= 7-13.

**Methods**

Blood was collected by cardiac puncture at the time of sacrifice. Sera were separated by centrifugation at 14,000 rpm for 15 minutes and stored at -80°C until assayed. A serum level of myostatin (GDF-8) was quantified using the commercially available GDF-8/Myostatin Immunoassay (DGDF80) and Human/Mouse/Rat Activin A Quantikine (DAC00B) ELISA kits by R&D systems Inc. (Minneapolis, MN), respectively. Samples and standards were performed in duplicate following the manufacturer’s instructions. The standard curve was generated by plotting the absorbance and concentration values of the standards using a four-parameter logistic curve-fit (online data analysis tool, MyAssays Ltd.) according to the manufacturer’s instruction.
