# Supplementary material for: Whole‐Body Metabolism and the Musculoskeletal Impacts of Targeting Activin A and Myostatin in Severe Osteogenesis Imperfecta
Source: JBMR Plus. 2023 May 7;7(7):e10753. doi: 10.1002/jbm4.10753 (PMC10339096; doi:10.1002/jbm4.10753)
Supplement: Supplementary file 2 — Fig. S2. Heart and spleen weights of Wt and oim/oim mice treated twice weekly with 10 mg/kg of control antibody (Ctrl‐Ab, black circle), anti‐activin A antibody (ActA‐Ab, blue triangle), anti‐myostatin antibody (Mstn‐Ab, red triangle), or combination anti‐activin A and anti‐myostatin antibodies (Combo, green square) from 5 to 16 weeks of age. (A) Absolute heart weight (mg), (B) relative heart weight (mg/g), (C) absolute spleen weight (mg), and (D) relative spleen weight (mg/g). Data represent min and max box and whisker plot with all data points shown; n = 8–13 mice per group; p‐values ≤ 0.1 are indicated and p ≤ 0.05 is considered significant. [file JBM4-7-e10753-s005.docx]

**Supplement Figure 2:** Heart and spleen weights of Wt and *oim/oim* mice treated twice weekly with 10mg/kg of either control antibody (Ctrl-Ab, black circle); anti-activin A antibody (ActA-Ab, blue triangle); anti-myostatin antibody (Mstn-Ab, red triangle) or combination anti-activin A and anti-myostatin antibodies (Combo, green square) from 5-16 weeks of age. A) Absolute Heart weight (mg), B) Relative Heart weight (mg/g), C) Absolute Spleen weight (mg), and D) Relative Spleen weight (mg/g). Data represent min and max box and whisker plot with all data points shown; n=8-13 mice per group; *p-values* ≤0.1 are indicated and p≤0.05 are considered significant.
