# Supplementary material for: Whole‐Body Metabolism and the Musculoskeletal Impacts of Targeting Activin A and Myostatin in Severe Osteogenesis Imperfecta
Source: JBMR Plus. 2023 May 7;7(7):e10753. doi: 10.1002/jbm4.10753 (PMC10339096; doi:10.1002/jbm4.10753)
Supplement: Supplementary file 4 — Fig. S4. Indirect calorimetry to assess energy status of Ctrl‐Ab‐, ActA‐Ab‐, Mstn‐Ab‐, and Combo‐treated Wt and oim/oim mice. (A) Mean energy expenditure (day cycle, kcal/h), (B) mean energy expenditure (night cycle, kcal/h), (C) mean O2 consumption (day cycle, mL/min), (D) mean O2 consumption (night cycle, mL/min), (E) mean CO2 production (day cycle, mL/min), (F) mean CO2 production (night cycle, mL/min), (G) mean respiratory quotient (day cycle), and (H) mean respiratory quotient (night cycle). Mice were treated twice weekly with 10 mg/kg of control antibody (Ctrl‐Ab, black circle), anti‐activin A antibody (ActA‐Ab, blue triangle), anti‐myostatin antibody (Mstn‐Ab, red triangle), or combination anti‐activin A and anti‐myostatin antibodies (Combo, green square) from 5 to 16 weeks of age. Data represent min and max box and whisker plot with all data points shown; n = 2–6. p‐values ≤ 0.1 are indicated, and p ≤ 0.05 is considered significant. [file JBM4-7-e10753-s002.docx]

**Supplement Figure 4:** Indirect calorimetry to assess energy status of Ctrl-Ab, ActA-Ab, Mstn-Ab and Combo-treated Wt and *oim/oim* mice. A) Mean energy expenditure (day cycle, kcal/hr), B) Mean energy expenditure (night cycle, kcal/hr), C) Mean O_2_ Consumption (Day cycle, ml/min), D) Mean O_2_ Consumption (Night cycle, ml/min), E) Mean CO_2_ Production (Day cycle, ml/min), F) Mean CO_2_ Production (Night cycle, ml/min), G) Mean Respiratory Quotient (Day Cycle), and H) Mean Respiratory Quotient (Night Cycle). Mice were treated twice weekly with 10mg/kg of control antibody (Ctrl-Ab, black circle); anti-activin A antibody (ActA-Ab, blue triangle); anti-myostatin antibody (Mstn-Ab, red triangle) or combination anti-activin A and anti-myostatin antibodies (Combo, green square) from 5-16 weeks of age. Data represent min and max box and whisker plot with all data points shown; n=2-6*. p-values ≤0.1* are indicated and *p-values ≤0.05* are considered significant.
