# Supplementary material for: Whole‐Body Metabolism and the Musculoskeletal Impacts of Targeting Activin A and Myostatin in Severe Osteogenesis Imperfecta
Source: JBMR Plus. 2023 May 7;7(7):e10753. doi: 10.1002/jbm4.10753 (PMC10339096; doi:10.1002/jbm4.10753)
Supplement: Supplementary file 5 — Fig. S5. Activity levels of Ctrl‐Ab‐, ActA‐Ab‐, Mstn‐Ab‐, and Combo‐treated Wt and oim/oim mice. Multidimensional beam breaks during (A) day and (B) night cycles are calculated from the averages of movement along the x‐, y‐, and z‐axes. Mice were treated twice weekly with 10 mg/kg of control antibody (Ctrl‐Ab, black circle) or a combination of activin A and myostatin antibodies (Combo, green square) from 5 to 16 weeks of age. Data represent min and max box and whisker plot with all data points shown; n = 5–10; p‐values ≤ 0.1 are indicated, and p ≤ 0.05 is considered significant. [file JBM4-7-e10753-s001.docx]

**Supplemental Figure 5:** Activity levels of Ctrl-Ab, ActA-Ab, Mstn-Ab and Combo-treated Wt and *oim/oim* mice.  Multidimensional beam breaks during the A) Day and B) Night cycles are calculated from the averages of movement along the X-, Y-, and Z-axes. Mice were treated twice weekly with 10mg/kg of control antibody (Ctrl-Ab, black circle); or a combination of activin A and myostatin antibodies (Combo, green square) from 5-16 weeks of age. Data represent min and max box and whisker plot with all data points shown; n=5-10; *p-values ≤0.1* are indicated and *p-values ≤0.05* are considered significant.
